# Supplementary material for: Foliar Infiltration of Virus-Derived Small Hairpin RNAs Triggers the RNAi Mechanism against the Cucumber Mosaic Virus
Source: Int J Mol Sci. 2022 Apr 29;23(9):4938. doi: 10.3390/ijms23094938 (PMC9104436; doi:10.3390/ijms23094938)

Figure S1: Bio-assays design. **Bio-assay 1:** Three biological replicates for each treatment. **Bio-assay 2:** Three (RT-qPCR) and six (ELISA) biological replicates for each treatment.

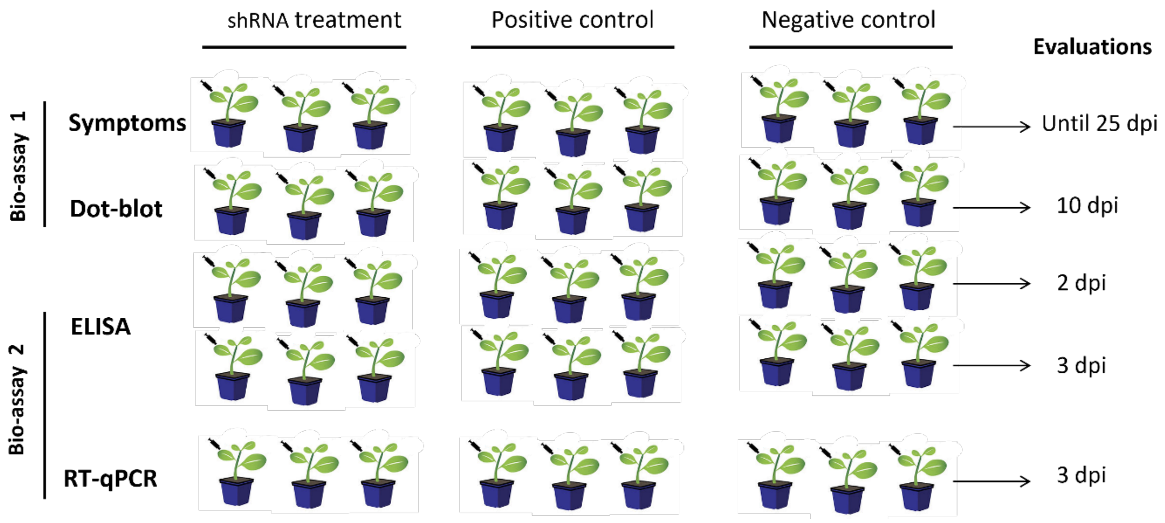

Supplement: Supplementary file 1 [file ijms-23-04938-s001.zip › ijms-1673965-supplementary.pdf]
